# m^6^A demethylase FTO regulate *CTNNB1* to promote adipogenesis of chicken preadipocyte

**DOI:** 10.1186/s40104-022-00795-z

**Published:** 2022-12-02

**Authors:** Kan Li, Weichen Huang, Zhijun Wang, Qinghua Nie

**Affiliations:** 1 Department of Animal Genetics, Breeding and Reproduction, College of Animal Science, South China Agricultural University, Guangzhou, 510642 China; 2 National-Local Joint Engineering Research Center for Livestock Breeding, Guangdong Provincial Key Lab of Agro-Animal Genomics and Molecular Breeding, and Key Laboratory of Chicken Genetics, Breeding and Reproduction, Ministry of Agriculture, Guangzhou, 510642 China

**Keywords:** Adipogenesis, Chicken, *CTNNB1*, FTO, m^6^A

## Abstract

**Background:**

N6-methyladenosine (m^6^A) is an abundant post-transcriptional RNA modification that affects various biological processes. The fat mass and obesity-associated (FTO) protein, a demethylase encoded by the *FTO* gene, has been found to regulate adipocyte development in an m^6^A-dependent manner in multiple species. However, the effects of the m^6^A methylation and FTO demethylation functions on chicken adipogenesis remain unclear. This study aims to explore the association between m^6^A modification and chicken adipogenesis and the underlying mechanism by which FTO affects chicken preadipocyte development.

**Results:**

The association between m^6^A modification and chicken lipogenesis was assessed by treating chicken preadipocytes with different doses of methyl donor betaine and methylation inhibitor cycloleucine. The results showed that betaine significantly increased methylation levels and inhibited lipogenesis, and the inverse effect was found in preadipocytes after cycloleucine treatment. Overexpression of *FTO* significantly inhibited m^6^A levels and promoted proliferation and differentiation of chicken preadipocytes. Silencing *FTO* showed opposite results. Mechanistically, *FTO* overexpression increased the expression of catenin beta 1 (*CTNNB1*) by improving RNA stability in an m^6^A-dependent manner, and we proved that FTO could directly target *CTNNB1*. Furthermore, *CTNNB1* may be a positive regulator of adipogenesis in chicken preadipocytes.

**Conclusions:**

m^6^A methylation of RNA was negatively associated with adipogenesis of chicken preadipocytes. FTO could regulate *CTNNB1* expression in a demethylation manner to promote lipogenesis.

**Supplementary Information:**

The online version contains supplementary material available at 10.1186/s40104-022-00795-z.

## Background

The prevalence of obesity has increased rapidly worldwide in recent years. Being overweight can lead to severe physical and psychological health problems, such as diabetes, cardiovascular disease and certain cancers [1]. Obesity even increases the risk of severe illness in patients with COVID-19 [2]. The association of obesity with different diseases makes it a growing public health issue [3].

After long-term genetic selection, commercial broilers with a faster growth rate were used for intensive production [4]. The mass of fat deposited accompanies the growth rate of the broiler, so excessive fat deposit usually occurs in the modern poultry industry [5]. Excessive fat deposition reduces feed efficiency and carcass nutritional value, increases farming costs and even affects consumer health. Reducing excess fat deposition has become an urgent problem in broiler production. Notably, due to their natural hyperglycemia and insulin resistance, chickens are often considered an ideal experimental animal model for the study of type 2 diabetes and obesity [6]. Therefore, studies on the regulatory mechanism of chicken fat deposition can provide some new insights for human biomedicine.

Adipogenesis is a complex process that involves the commitment of mesenchymal stem cells, the proliferation of preadipocytes, and the accumulation of lipid droplets within adipocytes [7]. The widely studied transcription factors CCAAT enhancer-binding proteins (C/EBPs), peroxisome proliferator-activated receptor-γ (PPARγ) and sterol regulatory element binding transcription factor 1 (SREBF1) are necessary for adipocyte development [8]. The expansion of adipose tissue is influenced by genetic, nutritional, and environmental factors [4]. Epigenetic regulation is also involved in various stages of adipogenesis [9]. m^6^A is the most common post-translational modification on eukaryotic RNA, widely present in tRNA, rRNA, mRNA, and non-coding RNA, accounting for more than 60% of all RNA modifications [10]. Currently, m^6^A has been shown to affect multiple processes such as mRNA splicing, export, translation, and degradation [11]. Methyltransferases (Writers), demethylases (Erasers), and methylated readers (Readers) are responsible for the addition, removal, and recognition of m^6^A modifications, respectively. Writers mainly include methyltransferase-like 3 (METTL3) and METTL14, and Wilms tumor 1 associated protein (WTAP). Erasers are mainly composed of FTO and AlkB homolog 5 (ALKBH5). m^6^A reader proteins mainly consist of YT521B homology families (YTHDF1/2/3), YTH domain families (YTHDC1/2), and IGF2BP protein family (IGF2BP1/2/3) [12]. The specific motif-containing RRACH (R = A or G; H = A, C or U) site was the main base sequence on RNA that acted with methyltransferase complex. Three m^6^A-related proteins, FTO, METTL3 and YTHDF2, have all been reported to be involved in adipogenesis [13, 14].

Genome-wide associated studies (GWAS) showed that genetic variants of the *FTO* gene were associated with obesity in multiple populations [15–17]. The association of *FTO*’s sequence variation with chicken fat mass and weight was also confirmed in our previous studies [18]. *FTO* ablation resulted in a reduction in fat mass and body weight [19, 20], and *FTO* overexpression increased food intake and fatness in mice [21]. In 2011, Prof. He Chuan's team first found that the knockout of *FTO* in human cells led to an increase in the global m^6^A level of mRNA [22]. *FTO* was found to regulate rodent adipogenesis by affecting alternative splicing of mRNA [23], RNA stability [24], and degradation [25] in an m^6^A-dependent manner. However, the regulatory role of *FTO* on chicken adipogenesis remains largely unknown.

This study investigated whether m^6^A modification regulates adipogenesis of chicken preadipocytes. Betaine and cycloleucine were added to chicken preadipocytes, and the results showed that the global m^6^A modification level was negatively correctly with adipogenesis of chicken preadipocytes. *FTO* overexpression markedly promoted the proliferation and differentiation ability of chicken preadipocytes. FTO directly targeted *CTNNB1* transcripts in an m^6^A-demethylation manner to regulate its mRNA expression, thereby affecting lipogenesis. The current study will provide new insights into the prevention and treatment of chicken excessive fat deposition.

## Methods

### Cell culture, differentiation and transfection

Immortalized chicken preadipocytes (ICP) were gifted from the Hui Li's lab of the College of Animal Science and Technology, Northeast Agricultural University. ICP cell was cultured in DMEM/F12 medium (Gibco, CA, United States) supplemented with 15% fetal bovine serum (Gibco, CA, United States) and 1% penicillin–streptomycin (Gibco, CA, United States) at 37 ℃ with 5% CO_2_. When the cell density reached 80%–90%, cells were digested and passaged with 0.25% trypsin (Gibco, CA, United States); and when the cell reached about 95%–100% confluency for adipocyte differentiation, the growth medium was replaced with a differentiation medium (DMEM/F12 medium, 15% fetal bovine serum, 1% penicillin–streptomycin, and 160 μmol/L sodium oleate (Merck, NJ, United States). Lipofectamine 3000 (Thermo Fisher, MA, United States) was used for the transfection of plasmids and oligonucleotides according to the instructions.

### Plasmid construction and oligonucleotides synthesis

*FTO* and *CTNNB1* sequence information were obtained from the NCBI website (the accessing number: NC_052542.1 and NC_052533.1). Specific primers were designed using oligo7 software (version 7.56, Molecular Biology Insights, US) and synthesized by Beijing Tsingke Biotechnology Co., Ltd. The full-length sequence of *FTO* and *CTNNB1* were amplified using high-fidelity enzymes (TransGen, Beijing, China) and ligated into the pcDNA3.1 or pcDNA3.1 3 × Flag-C vector (YuoBio, Changsha, China). Shanghai GenePharma Co., Ltd. provided the design and synthesis of siRNA. The primer information and oligonucleotide were shown in (Additional file 1: Table S1 and Table S2).

### RNA extraction and quantitative real-time PCR

According to the instructions, total RNA was extracted and purified using Trizol Reagent (Accurate Biology, Wuhan, China). RNA concentration and integrity were determined using NanoDrop one spectrophotometer. MonScript™ RTIII All-in-One Mix with dsDNase (Monad, Shanghai, China) kit used for cDNA transcription. Real-time quantitative PCR (qPCR) assay was performed using ChamQ Universal SYBR qPCR Master Mix (Vazyme, Nanjing, China). The reaction system is 1 μL cDNA, 5 μL ChamQ Universal SYBR qPCR Master Mix, 0.3 μL upstream and downstream primers, and 3.4 μL ddH_2_O. The reaction mixture was run at the following program: 95 °C, 3 min; (95 °C, 20 s; 58 ℃, 30 s) for 40 cycles; dissolution curve: 55 °C warm up to 95 °C, collect signal every 0.5 °C. According to the 2^−ΔΔCt^ algorithm, the relative expression level was calculated, in which β-actin was used for internal reference. qPCR primers were designed using oligo7 software (Additional file 1: Table S1 for primers).

### Phylogenetic tree construction and protein functional domain analysis

Amino acid sequences of FTO in different species were acquired using the NCBI database. These accessing numbers were as follows: NP_001172076.1 (chicken), NP_001073901.1 (human), NP_036066.2 (mouse), NP_001034802.1 (Norway rat), NP_001106162.1 (pig), NP_001091611.1 (cattle), XP_014981634.1 (rhesus monkey), XP_027322973.1 (mallard), NP_001098401.1 (sheep), XP_019475299.1 (turkey), and XP_015729304.1 (quail). A phylogenetic tree was drawn by the Neighbor-joining statistical method and Bootstrap detection method using MEGA software (version 11; available at: http://www.megasoftware.net). The identification and annotation of the FTO protein domain were analyzed using an online tool called SMART (simple modular architecture research tool; available at: http://smart.embl-heidelberg.de/).

### Western blotting

Cell protein extraction was performed according to the instructions of the RIPA buffer (Beyotime, Shanghai, China). Briefly, cells were washed once with pre-cold PBS buffer and lysed on ice for 30 min using RIPA buffer pre-added with PMSF (Beyotime, Shanghai, China). The lysate supernatant was collected by centrifugation at 12,000 × *g* at 4 °C for 10 min. SDS-PAGE sample loading buffer (Beyotime, Shanghai, China) was added to supernatant for protein denaturation in a preset metal water bath of 95 °C. The proteins were separated by SDS-PAGE gel and transferred to the PVDF membrane using the wet transfer method. The membrane was blocked in 5% skim milk powder solution for 1 h. The diluted primary antibody solution was incubated with PVDF membrane overnight at 4 °C. The membrane was then washed three times with TBST for 5 min. The PVDF membrane was incubated in the secondary antibody solution for 1 h at room temperature, after which the membrane was washed three times with TBST for 5 min. PVDF membranes were incubated in ECL substrate solution for 5 min and exposed and imaged using the Odyssey-Fc system (Licon, Germany). Antibody information was shown as follows: rabbit anti-FTO (Novus, CO, United States), rabbit anti-Flag (Proteintech, Wuhan, China), rabbit anti-PPARγ (Bioss, Beijing, China), rabbit anti-C/EBPβ (Bioss, Beijing, China), rabbit anti-β-catenin (Bioss, Beijing, China), mouse anti-GAPDH (Bioss, Beijing, China), goat anti-rabbit IgG H&L antibody (Bioss, Beijing, China), goat anti-mouse IgG H&L antibody (Bioss, Beijing, China).

### RNA dot blot

Total RNA was denatured at 95 °C for 3 min and then immediately placed on ice to prevent RNA secondary structure re-formation. The denatured RNA was dropped on the positively charged nylon membrane, and the RNA was cross-linked to the nylon membrane using a UV cross-linker (2000 J for 1 min). Wash the nylon membrane once with TBST for 5 min. Afterward, the methylation level was detected by the specific m^6^A antibody (CST, MA, United States) according to the western blot method. The nylon membrane was transferred to a dish containing methylene blue staining buffer, and incubated for 30 min with gentle shaking. Tap water is used to rinse the membrane until the background is clean. The images were inquired using Tanon 2500 Gel Image Analysis System (Tanon, Shanghai, China).

### Cell counting Kit 8 (CCK-8) assay

The CCK8 reagent (Beyotime, Shanghai, China) was used to detect cell proliferation. Cells in the exponential growth phase were seeded in 96-well plates at a density of 1 × 10^3^. Transfection was performed when the cell density reached 60%–70%, and cell proliferation was detected 12, 24, 36, and 48 h after transfection. Before detection, 10 μL of CCK8 reagent was added to each well, and after incubation at 37 °C for 1.5 h, the absorbance at 450 nm wavelength was detected using a Model 680 Microplate Reader (Bio-Rad, CA, United States).

### 5-Ethynyl-2’-deoxyuridine (EdU) assay and flow cytometry EdU assay

The EdU assay was performed according to the instructions of the Cell-Light EdU Apollo488 In Vitro Kit (RiboBio, Guangzhou, China). Briefly, the transfected cells were incubated with 10 μmol/L EdU solution for 2 h. The 4% paraformaldehyde solution was used to fix cells for 30 min, and then the cells were permeabilized with 0.5% Triton X-100 solution for 30 min. Subsequently, the cells were incubated with Apollo reaction solution for 30 min in the dark. Finally, the cells were washed three times with PBS buffer containing 0.5% Triton X-100 solution for 10 min and incubated with Hoechst reaction buffer for 30 min. The images were acquired and analyzed using fluorescence microscope DMi8 (Leica, Wetzlar, Germany) and image J software (National Institutes of Health). The flow cytometry EDU assay is similar to those described above, except that cells are collected and resuspended in tubes for subsequent fixation and staining. Finally, the fluorescently stained cells were analyzed by flow cytometry (BD, NJ, United States).

### Oil red O staining

The adipogenic differentiated cells were washed twice with PBS buffer and then fixed in 10% formalin for 1 h. Next, the fixed cells were washed twice with water, rinsed with 60% isopropanol for 5 min, and dried completely at RT. The cells were stained with an Oil red O working solution for 10 min and afterward washed four times with water. The images were acquired under the microscope DMi8 for analysis. Oil red O dye was eluted 2–5 times with 100% isopropanol to ensure that all Oil red O dye was in the solution. The solution was transferred to 96-well plates. The OD values at 500 nm were detected using a Fluorescence/Multi-Detection Microplate Reader (BioTek, Winooski, VT, USA). The results of the oil red extraction assay were normalized using total protein content in adjacent wells with the same treatment.

### Nile red staining

In brief, adipogenic differentiated cells were washed twice with PBS solution and fixed with 4% paraformaldehyde for 30 min, then washed twice with PBS. The cells were stained with Nile red solution (Beyotime, Shanghai, China) for 10 min in the dark, and after that, DAPI solution (Beyotime, Shanghai, China) was used to stain for 2 min under dark conditions. Fluorescent images were obtained using fluorescence microscope DMi8. The fluorescence intensity and the number of nuclei were analyzed using Image J software. The results were normalized using the ratio of fluorescence intensity of Nile red to the number of nuclei of DAPI.

### Single-base elongation and ligation-based PCR amplification method (SELECT)

The SELECT method was the previously described [26]. Briefly, total RNA was mixed with 40 nmol/L upstream primer, 40 nmol/L downstream primer, 5 µmol/L dNTP, and 1.7 μL 10 × CutSmart buffer into a total reaction solution of 17 μL. The RNA and primers were annealed as the following procedure: 90 ℃ for 1 min, 80 ℃ for 1 min, 70 ℃ for 1 min, 60 ℃ for 1 min, 50 ℃ for 1 min, 40 ℃ for 6 min. Subsequently, the first step reaction solution added 3 μL of a mixture consisting of 0.01 U Bst 2.0 DNA polymerase, 0.5 U SplintR ligase, and 10 nmol/L ATP. The single base ligation procedure is 40 ℃ for 20 min and 80 ℃ for 20 min. Finally, CT values were then compared using real-time PCR as described above. Specific upstream and downstream primers were synthesized based on m^6^A predicted sites (Additional file 1: Table S3 for SELECT primers).

### Methylated RNA immunoprecipitation assay

A total amount of 5 μg of RNA was incubated with RNA fragmentation buffer (100 mmol/L Tris–HCl, 100 mmol/L ZnCl_2_) in a thermal cycler at 70 °C for 5 min and fragmented into about 200 nt in size. m^6^A antibody (CST, MA, United States) was incubated with pre-washed protein A/G magnetic beads (MCE, Shanghai, China) overnight at 4 °C. The magnetic bead-antibody mixture was separated and adsorbed to the side of the tube using a magnetic separation rack and washed twice with IP buffer (20 mmol/L Tris–HCl pH = 7.5, 150 mmol/L NaCl, 1.5 mmol/L MgCl_2_, 10% glycerin, 0.5% NP40, 0.5% Triton-X100). The mixture was resuspended in 500 μL of reaction solution (fragmented RNA, 100 μL 10 × IP buffer, and 400 U/μL RNase inhibitor) and incubated at 4 °C for 6–8 h. The magnetic bead-antibody RNA mixture was washed twice in IP buffer, then twice in low-salt solution (50 mmol/L NaCl, 10 mmol/L Tris–HCl pH = 7.5, 0.1% NP40), and twice in high-salt solution (500 mmol/L NaCl, 10 mmol/L Tris–HCl pH = 7.5, 0.1% NP40) at 4 ℃ for 5 min each. The m^6^A-enriched RNA was eluted and purified using the RNeasy MinElute Cleanup Kit (Qiagen, Hilden, Germany). Real-time PCR was used to assess the methylation level of the specific site.

### RNA-binding protein immunoprecipitation assay

Approximately 4 × 10^7^ cells are required for the total number of cells. Cells were washed once with pre-chilled PBS buffer and collected by cell scrapers into 50 mL RNase-free centrifuge tubes. The cell pellets were re-suspended and lysed with IP buffer containing PMSF and RNase inhibitor for 30 min on ice. The lysate supernatants were collected by centrifugation at 12,000 × *g* at 4 °C for 10 min. The protein A/G magnetic beads were incubated with an IP antibody (the dilution ratio is 100:1) overnight at 4 ℃. The lysates were incubated magnetic-antibody mixture for 6 h at 4 ℃. The reaction mixtures were centrifugated at 3000 r/min at 4 °C for 2 min. The supernatants were discarded, and then magnetic beads were washed 5 times with IP buffer. The magnetic beads were incubated with proteinase K-containing eluent in a metal water bath at 55 °C for 45 min. The supernatants were collected by centrifugation at 3000 r/min at 4 °C for 5 min. The RNA in supernatants was eluted and purified using the RNeasy MinElute Cleanup Kit (Qiagen, Hilden, Germany). Finally, the interaction between RNA and protein was determined using real-time PCR.

### RNA stability assay

The transfected cells were treated with actinomycin D (5 μg/mL) (MCE, shanghai, China). Total RNA was extracted at 0, 3, and 6 h to assess mRNA degradation with qPCR.

### Statistical analysis

GraphPad Prism 8.0 software (San Diego, CA, USA) was used for all image drawing and statistical analysis. Differences between two groups were analyzed using an unpaired two-tailed Student's *t*-test. ANVOA (analysis of variance) was used to evaluate differences between multiple groups and followed by Tukey's multiple comparisons test. Results were statistically significant when the *P*-value was less than 0.05. Data were expressed as mean ± standard error of the mean (SEM).

## Results

### Effects of global RNA m^6^A modification levels on the development of chicken preadipocytes

**Fig. 1:**
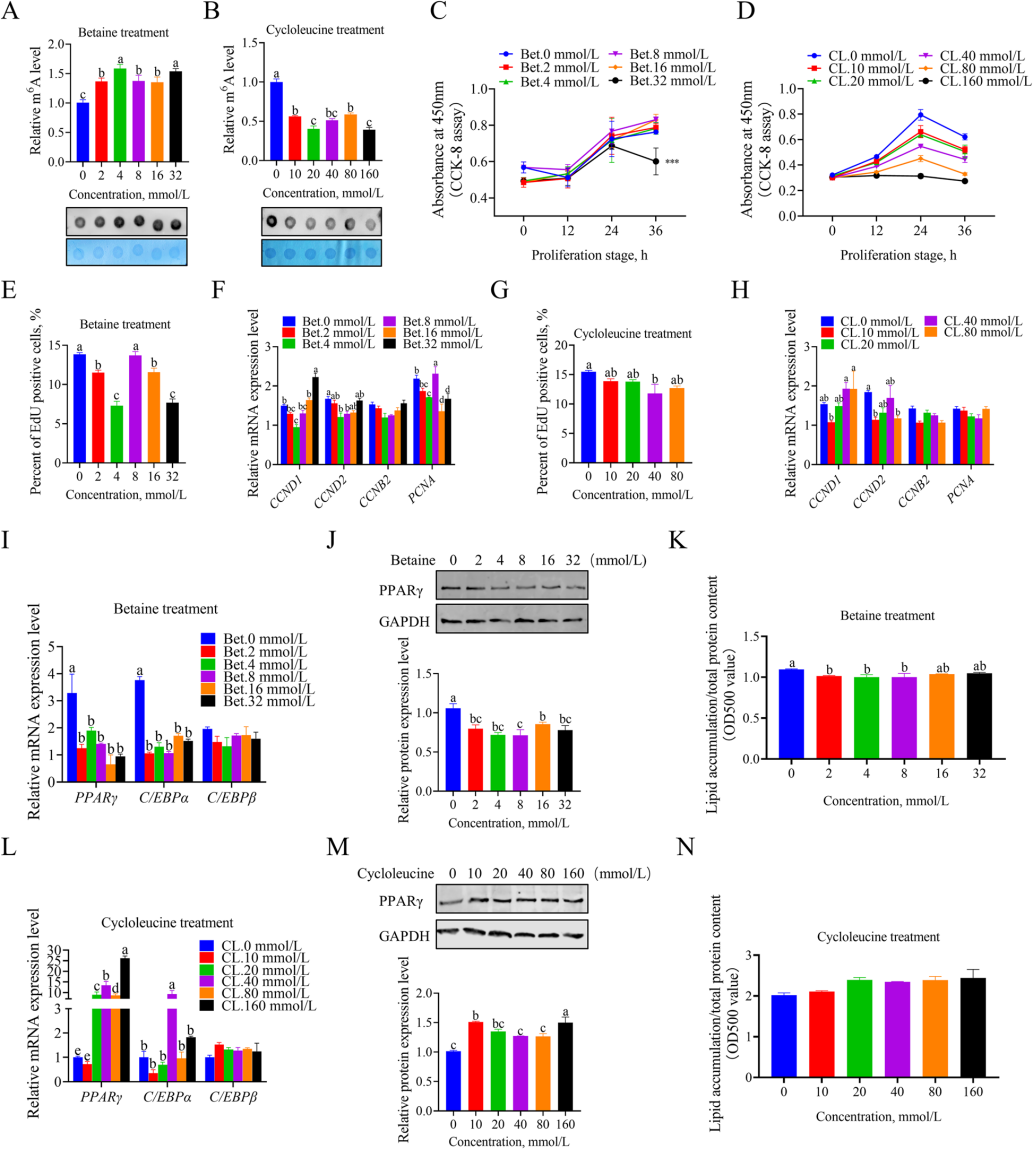
Betaine and cycloleucine supplementation affect global m^6^A levels and lipogenesis in chicken preadipocytes. **A** and **B** Detection of RNA m^6^A levels in chicken preadipocyte supplement with betaine and cycloleucine by RNA dot blot and methylene blue staining; **C** and **D** Cell proliferation rate by CCK8 treated with different doses of betaine and cycloleucine; **E** Cell proliferation analysis by flow cytometry EdU assay for betaine treatment; **F** The relative mRNA expression of proliferation-related genes of betaine treatment was detected in chicken preadipocyte; **G** Cell proliferation analysis by flow cytometry EdU assay for cycloleucine treatment; **H** The relative mRNA expression of proliferation-related genes of cycloleucine treatment was detected in chicken preadipocyte using qPCR; **I** The relative mRNA expression of adipogenic differentiation genes of betaine treatment was detected in chicken preadipocytes using qPCR; **J** The relative protein expression of PPARγ was detected by western blot in chicken preadipocytes treated with betaine; **K** The quantification of lipid droplet in chicken preadipocytes treated with different concentrations betaine; **L** The relative mRNA expression level of adipogenic differentiation genes of cycloleucine treatment was detected in chicken preadipocytes using qPCR; **M** The relative protein expression of PPARγ was detected by western blot in chicken preadipocytes treated with cycloleucine; **N** The quantification of lipid droplet in chicken preadipocytes treated with different concentrations betaine. The results of all groups are shown as mean ± SEM of at least three biological replicates. Statistical significances were assessed using one-way analysis of variance (ANOVA), followed by Tukey's multiple comparisons test. Groups marked with the same letter were considered to have no significant difference, and those without the same letter were significantly different

The formation of m^6^A on RNA refers to the addition of S-adenosylmethionine (SAM), the major methylation donor, to the sixth nitrogen atom of adenine under the catalysis of the methyltransferase complex. Betaine is a major methyl donor for organic biomolecules and increases cellular SAM levels [27]. Cycloleucine is a competitive inhibitor of S-adenosylmethionine transferase. This study first investigated the effects of betaine and cycloleucine on RNA m^6^A modification, proliferation and differentiation of chicken preadipocytes. RNA dot blot is a straightforward method to detect global RNA methylation levels. The results showed that betaine significantly increased the m^6^A level in chicken preadipocytes, while cycloleucine treatment inhibited RNA m^6^A methylation (Fig. 1A, B). The CCK8 assay was used to evaluate the proliferation status of the cell. There was no difference in the proliferation state of cells treated with different concentrations of betaine in different periods, except for the high concentration of 32 mmol/L (Fig. 1C). However, cycloleucine inhibited cell proliferation in a dose-dependent manner, cells treated with high concentration 160 mmol/L cycloleucine did not proliferate, and subsequent proliferation assays removed this concentration group. (Fig. 1D). The proliferation status of cells treated with the two chemicals for 24 h was then analyzed using flow EdU assay and RT-PCR. The specific effects of different doses of betaine treatment on the proliferation rates and expression level of proliferation-related genes are similar, and the overall effects were inhibited, and 4 mmol/L of betaine significantly inhibited proliferation status and the expression level of cyclin D1 (*CCND1*), cyclin D2 (*CCND2*) and proliferating cell nuclear antigen (*PCNA*) mRNA (Fig. 1E, F; Additional file 1: Fig. S1A). The flow cytometry EdU assay results were consistent with the CCK8 assay in different concentrations of cycloleucine treatment groups (Fig. 1G; Additional file 1: Fig. S1B). For RT-PCR results of proliferation genes, it was found that, except for *CCND1*, the expression results of others were consistent with those in the cell proliferation test (Fig. 1H). The effect of different concentrations of betaine and cycloleucine on the differentiation ability of chicken adipocytes was then examined. The mRNA expressions of adipogenic differentiation-related genes *PPARγ, c/EBPα* and *c/EBPβ* were decreased considerably treated with different doses of betaine (Fig. 1I), and betaine significantly reduced the protein expression level of PPARγ, an essential gene for adipogenicity (Fig. 1J). Oil red O and Nile red staining results also indicated that betaine reduced fat deposition in chicken preadipocytes (Fig. 1K; Additional file 1: Fig. S1C, S2A). On the contrary, the addition of cycloleucine significantly promoted the mRNA expression of critical genes in adipogenicity (Fig. 1L), and the protein expression of PPARγ also significantly increased (Fig. 1M). The lipid accumulation in different concentrations of the cycloleucine treatment group also increased compared with the control group (Fig. 1N; Additional file 1: Fig. S1D, S2B).

### Bioinformatics analysis of chicken FTO and its demethylation effect in chicken preadipocytes

**Fig. 2:**
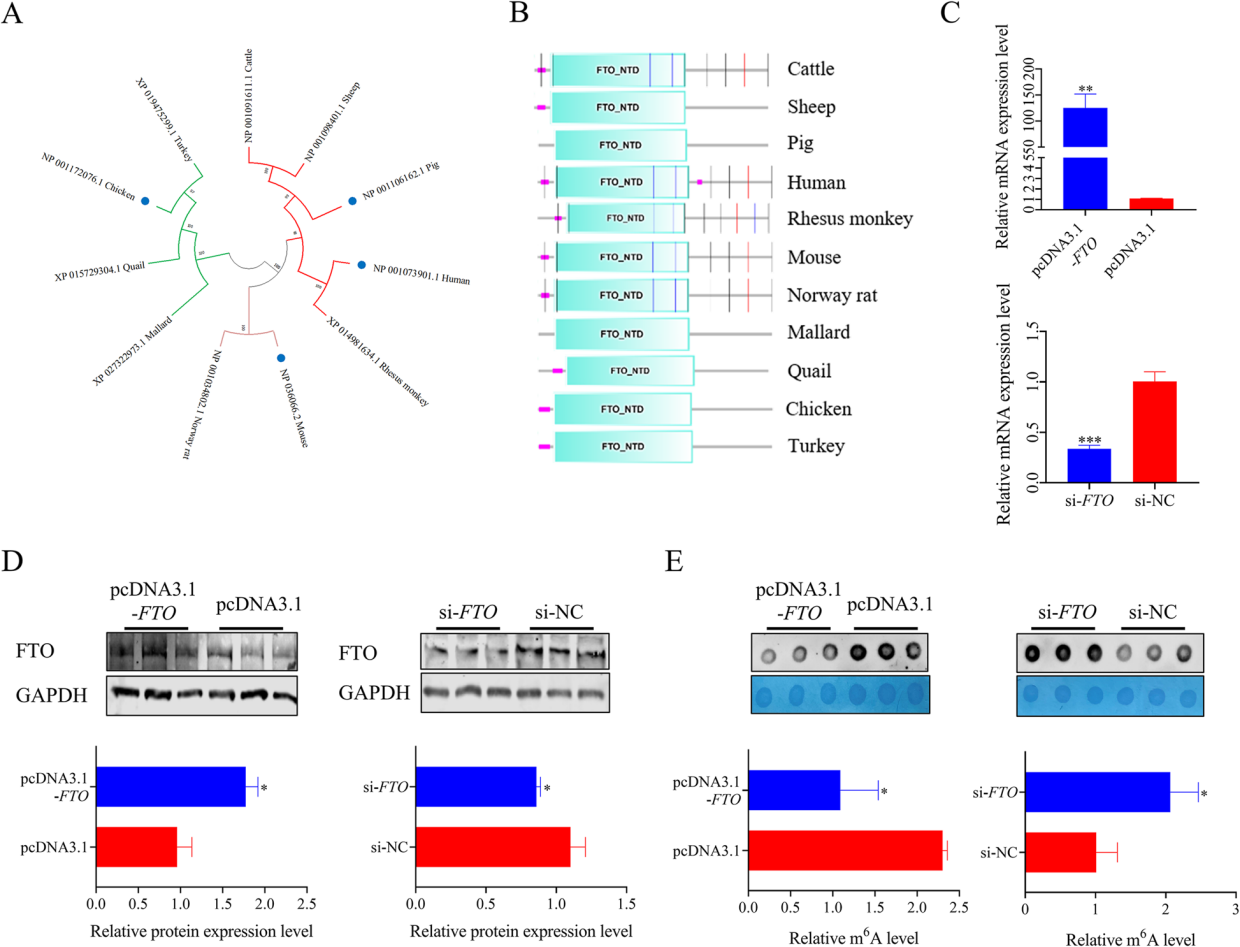
Bioinformation analysis and demethylation effects of chicken *FTO* gene. **A** Phylogenetic tree of the *FTO*; **B** Analysis and annotation of FTO protein domains among different species; **C** and **D** The relative mRNA and protein expression of FTO were detected after transfer with pcDNA3.1-*FTO* and siRNA in chicken preadipocytes using RT-PCR and western blotting; **E** RNA m^6^A levels in chicken preadipocytes were detected after transfection of pcDNA3.1-*FTO* and siRNA by RNA dot blot and methylene blue staining. These values are shown as mean ± SEM of at least three biological replicates. The statistical significance of the differences was assessed using the unpaired Student's *t*-test (* *P* < 0.05; **P < 0.01; ***P < 0.001)

The underlying mechanisms by which FTO regulates chicken preadipocyte development via demethylation are poorly understood. In this study, we first investigated and analyzed the homology of FTO proteins. A phylogenetic tree of the *FTO* gene was constructed using Mega 11.0 software (Fig. 2A). The results showed that the closest relative to the chicken is the turkey, and chicken is relatively distantly related to humans and mice, two species with proven FTO demethylation effects on adipocytes. Protein domains of 11 species were identified and analyzed using the SMART online tool. The FTO_NTD protein domain was present in all species selected for analysis (Fig. 2B). This domain is a catalytic AlkB-like domain from the FTO protein with demethylase activity. Next, overexpression plasmid and siRNA of *FTO* were constructed and transfected in preadipocytes to determine the effect of m^6^A modification of FTO in chicken. The qPCR results showed that the effects of overexpression and knockdown had reached a significant level (Fig. 2C). The protein level of FTO was similar to the qPCR results (Fig. 2D). The dot blot results showed that ectopic expression of *FTO* significantly reduced RNA m^6^A modification level. In contrast, the knockdown of *FTO* showed the opposite effect (Fig. 2E).

### *FTO* promote proliferation of chicken preadipocytes in vitro

**Fig. 3:**
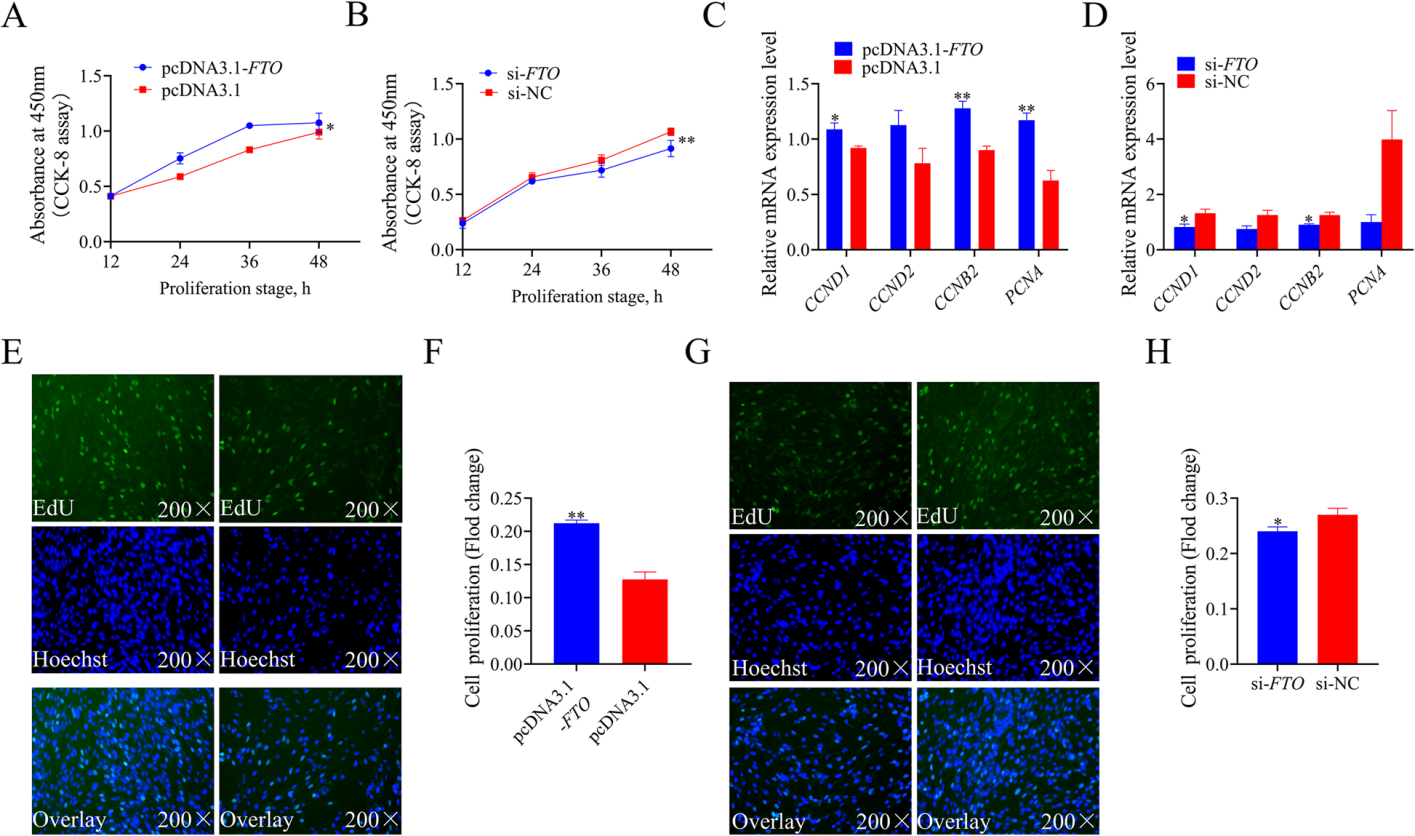
*FTO* promotes the proliferation of chicken preadipocytes. **A** and **B** CCK8 assay was used to detect cell proliferation rate with *FTO* overexpression and knockdown in chicken preadipocytes; **C** and **D** The relative mRNA expression of proliferation-related genes was detected after transfer with *FTO* overexpression plasmid and *FTO* siRNA in chicken preadipocytes using RT-PCR; **E** EdU staining assays for chicken preadipocyte transferred with pcDNA3.1-*FTO* and pcDNA3.1 for 48 h. **F** Fold change in proliferation rates of *FTO* overexpression using EdU assay. **G** EdU staining assays for chicken preadipocyte transferred with si-*FTO* and si-NC. **H** Fold change in proliferation rates of *FTO* knockdown using EdU assay. The values are shown as mean ± SEM of at least three biological replicates. The statistical significance of the differences was assessed using the unpaired Student's *t*-test (* *P* < 0.05; **P < 0.01)

To evaluate the role of *FTO* on the proliferation of chicken preadipocytes, *FTO* was overexpressed and knocked down by transfection of pcDNA3.1-*FTO* and siRNA, respectively. The CCK8 assay was used to detect the proliferation status of the cell at different times after transfection. The results showed that the number of viable cells significantly increased when *FTO* was overexpressed in preadipocytes (Fig. 3A), whereas knockdown of *FTO* significantly inhibited the cell proliferation ability (Fig. 3B). The qPCR analyses showed that these preadipocytes showed increased expression of *CCND1*, *CCND2*, Cyclin B2 (*CCNB2*), and *PCNA* in the *FTO*-overexpression conditions (Fig. 3C). The mRNA expression level of these proliferation-related genes was lower in *FTO* knockdown than in the control group (Fig. 3D). EdU assay also was used to detect and quantify cell proliferation. The results showed that the proliferation rate was significantly increased when *FTO* overexpression (Fig. 3E, F), whereas knockdown of *FTO* dramatically attenuated the proliferation rate (Fig. 3G, H).

### *FTO* regulate adipogenesis of chicken preadipocytes in vitro

**Fig. 4:**
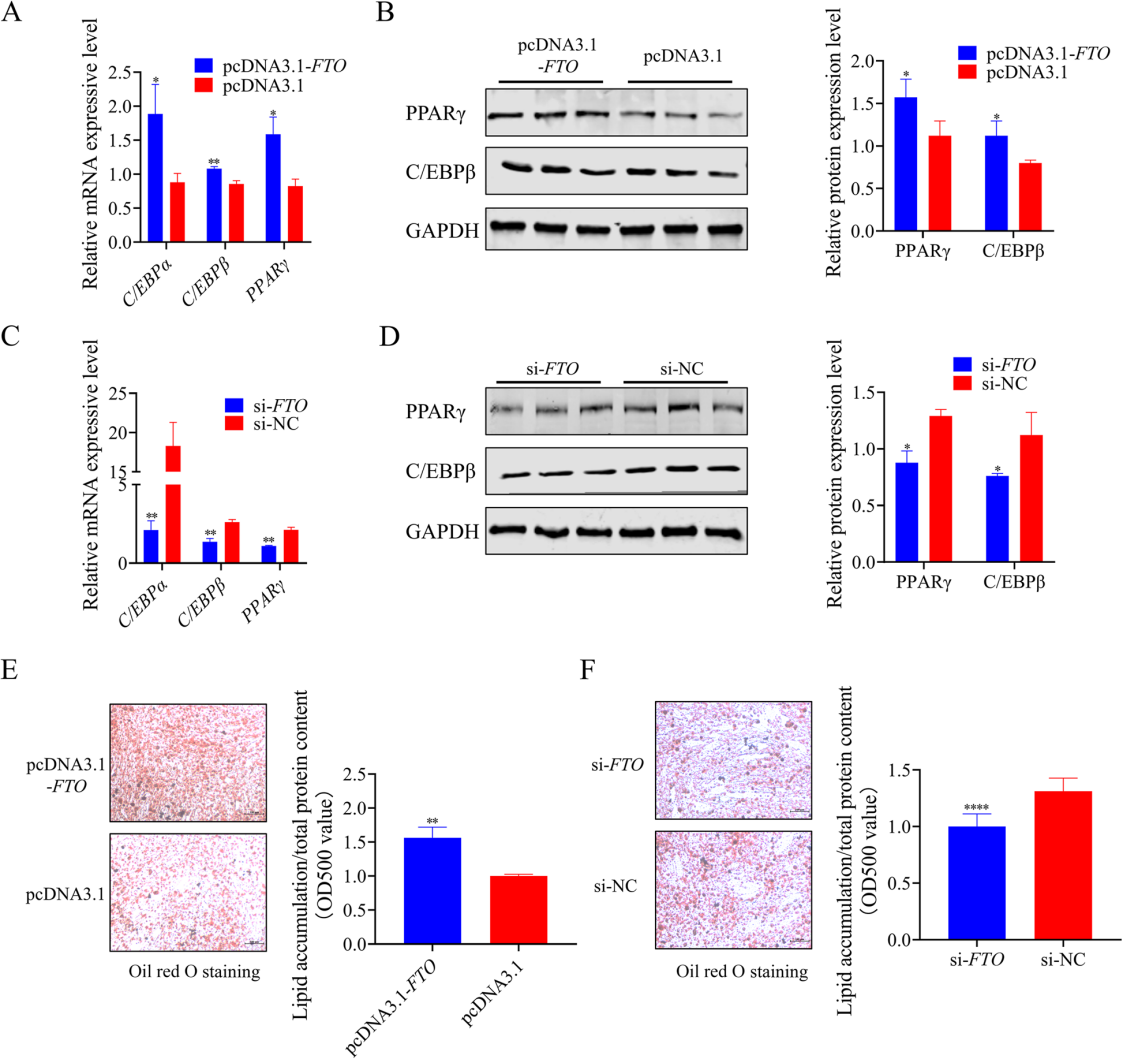
*FTO* promotes the adipogenesis of chicken preadipocytes. **A** and **B** The relative mRNA and protein levels of differentiation-related genes were detected in chicken preadipocytes transfected with overexpression plasmid of *FTO* using qPCR and western blot; **C** and **D** The relative mRNA and protein levels of differentiation-related genes were detected in chicken preadipocytes transfected with si-*FTO* and si-NC using qPCR and western blot; **E** Oil red O staining results and quantification of lipid droplet in chicken preadipocytes after 48 h transfection of *FTO* overexpression plasmid; **F** Oil red O staining results and quantification of lipid droplet in chicken preadipocytes after 48 h transfection of *FTO* siRNA. These values are shown as mean ± SEM of at least three biological replicates. The statistical significance of the differences was assessed using the unpaired Student's *t*-test (* *P* < 0.05; **P < 0.01; ****P < 0.0001)

To determine the adipogenic capacity of *FTO*, we next examined the transcriptional and protein expression levels of adipogenesis-related genes after adipogenic induction. Transfection of *FTO* overexpression plasmid significantly promoted the expression of *C/EBPα*, *C/EBPβ*, and *PPARγ*, three critical genes that are important for regulating adipogenesis (Fig. 4A), and overexpression of *FTO* led to a significant upregulation of both PPARγ and C/EBPβ protein level (Fig. 4B). In contrast, the mRNA expression levels of *C/EBPα*, *C/EBPβ*, and *PPARγ* were significantly suppressed in *FTO* knockdown cells compared with that control (Fig. 4C), and similar changes in protein expression levels were also found (Fig. 4D). The lipid content was visualized and quantified using Oil red O staining and a spectrophotometer. There were more Oil red O positive cells after adipogenic induction in *FTO* overexpression preadipocytes, and the value of absorbance also showed more significant triglyceride accumulation than controls (Fig. 4E). Knockdown of *FTO* showed lower lipid droplet formation than the control group (Fig. 4F).

### FTO mediated the demethylation of *CTNNB1*

**Fig. 5:**
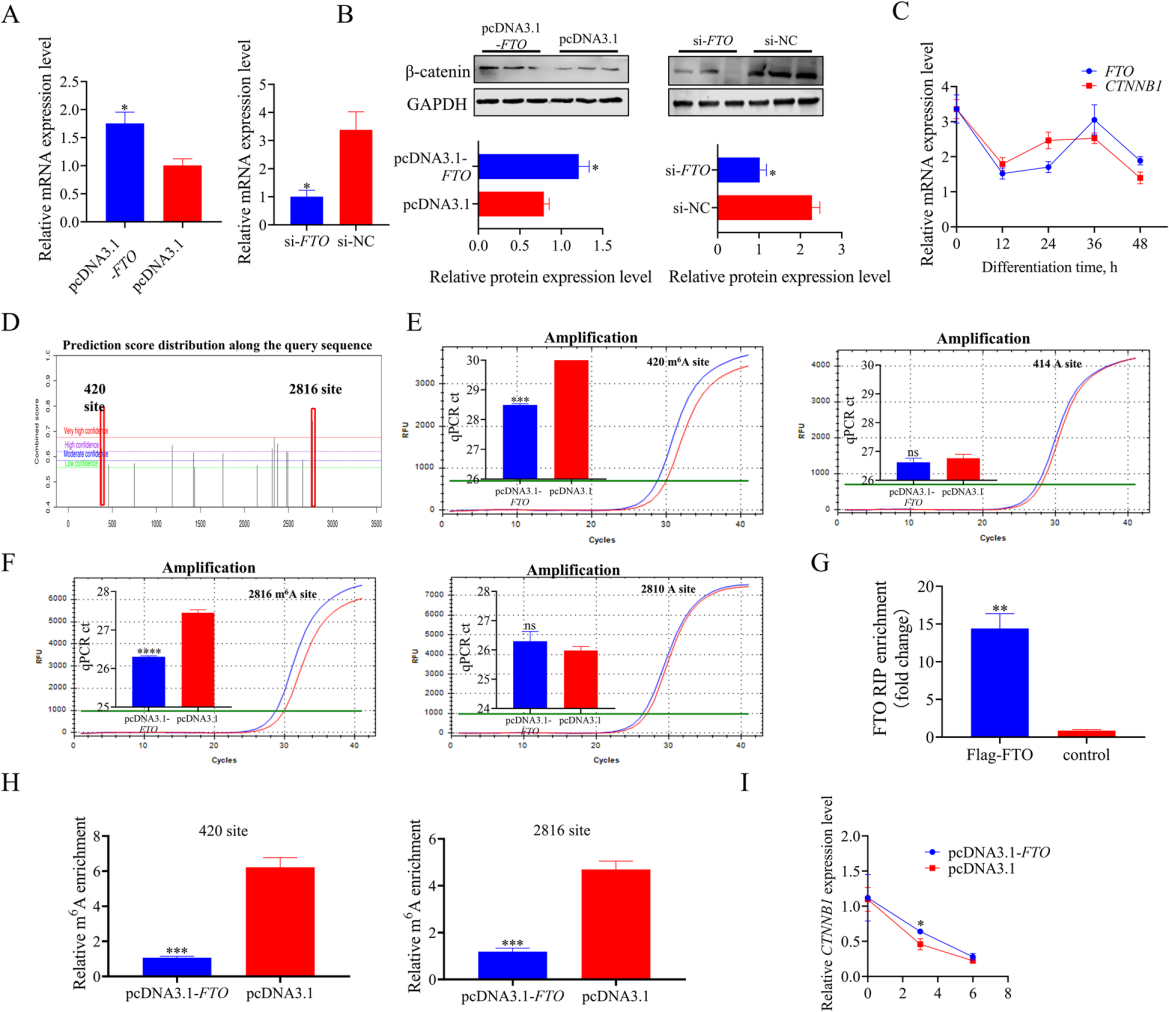
*FTO* regulate *CTNNB1* expression in m^6^A depend manner. **A** The relative mRNA expression of *CTNNB1* was detected after *FTO* overexpression or knockdown using qPCR; **B** The relative protein expression of β-catenin were detected after *FTO* overexpression or knockdown using western blot; **C** The expression profile of *FTO* and *CTNNB1* during chicken preadipocyte differentiation process; **D** Prediction of m^6^A modification sites in mature *CTNNB1* mRNA using online tool SRAMP; **E** Amplification curve and qPCR CT value in *CTNNB1* 420 m^6^A site and 414 A site after *FTO* overexpression; **F** Amplification curve and qPCR CT value in *CTNNB1* 2816 m^6^A site and 2810 A site after *FTO* overexpression; **G** RIP assays were performed to verify the interaction with FTO protein and *CTNNB1* mRNA; **H** MeRIP assays were applied to assess that the m^6^A methylation level changes after *FTO* overexpression in chicken preadipocytes; **I** The RNA stability of *CTNNB1* mRNAs was detected by qPCR with *FTO*-overexpressing in chicken preadipocytes after treatment of Act D. These values are shown as mean ± SEM of at least three biological replicates. The statistical significance of the differences was assessed using the unpaired Student's *t*-test (* *P* < 0.05; ***P < 0.001; ****P < 0.0001)

The protein β-catenin encoded by the *CTNNB1* gene is a crucial regulator of the canonical wnt/β-catenin signaling pathway regulating adipocyte development. To further assess the potential role of *CTNNB1* in *FTO*-regulated chicken obesity development, we examined the expression of *CTNNB1* in *FTO*-overexpressed and knockdown chicken preadipocytes. *CTNNB1* expression was significantly higher in preadipocytes overexpressing *FTO* than in controls, and knockdown of *FTO* significantly decreased *CTNNB1* expression (Fig. 5A, B). Moreover, we examined the expression profile of *FTO* and *CTNNB1* during chicken preadipocyte differentiation process and found that the expression patterns of the two were positively correlated (Fig. 5C). To further explore whether FTO regulates changes in *CTNNB1* expression levels in a demethylation-dependent manner, we next predicted and analyzed all possible m^6^A modification sites in the mature *CTNNB1* mRNA sequence by the online tool SRAMP [28] and previous reports [29] (Additional file 1: Table S4). Site 420 in the coding region and 2816 in the 3'-UTR area were identified as candidate methylation sites (Fig. 5D). Next, we analyzed the probability of interaction between FTO protein and mature *CTNNB1* mRNA sequence using online RNA–protein interaction prediction (RPISeq) [30] and protein-RNA interaction predictor (PRIdictor) [31]. These bioinformatic analyses indicated that both had a positive binding ability (Table 1; Additional file 1: Fig. S3). We performed further validation using the SELECT method to confirm the possible association between changes in site-specific m^6^A modification level of *CTNNB1* and FTO protein. The N-site at 6 bases upstream of the predicted site was set as a negative control. qPCR results showed that the m^6^A modification levels at sites 420 and 2816 were significantly reduced in *FTO*-overexpression cells compared with the controls, and no significant difference was observed in the amplification of sites 414 and 2810 (Fig. 5E, F). The opposite effect was found in *FTO* knockdown preadipocytes (Additional file 1: Fig. S4). We next transferred the pcDNA3.1-*FTO* 3 × Flag-C vector into chicken preadipocytes and then performed a RIP assay to confirm the interaction between FTO and *CTNNB1*. The results showed that *CTNNB1* could be pulled down by the Flag antibody (Fig. 5G). MeRIP-qPCR analysis also demonstrated that the m^6^A level of *CTNNB1* at sites 420 and 2816 was decreased in *FTO*-overexpression preadipocytes (Fig. 5H). The stability of *CTNNB1* was assessed after actinomycin D treatment in *FTO* overexpression preadipocytes. The results showed that decreased m^6^A methylation level could increase *CTNNB1* stability (Fig. 5I).

**Table 1:** RNA–Protein interaction prediction (RPISeq)

Protein ID	RNA ID	Interactive probabilities	
		**Prediction using RF classifier**	**Prediction using SVM classifier**
> NP_001172076.1 alpha-ketoglutarate-dependent dioxygenase FTO (*Gallus gallus*)	> NM_205081.3 Gallus gallus catenin beta 1 (*CTNNB1*), mRNA	0.8	0.94

Interaction probabilities generated by RPISeq range from 0 to 1. In performance evaluation experiments, predictions with probabilities > 0.5 were considered “positive," that is, indicating that the corresponding RNA and protein are likely to interact

### *CTNNB1* promotes lipid accumulation of chicken preadipocytes

**Fig. 6:**
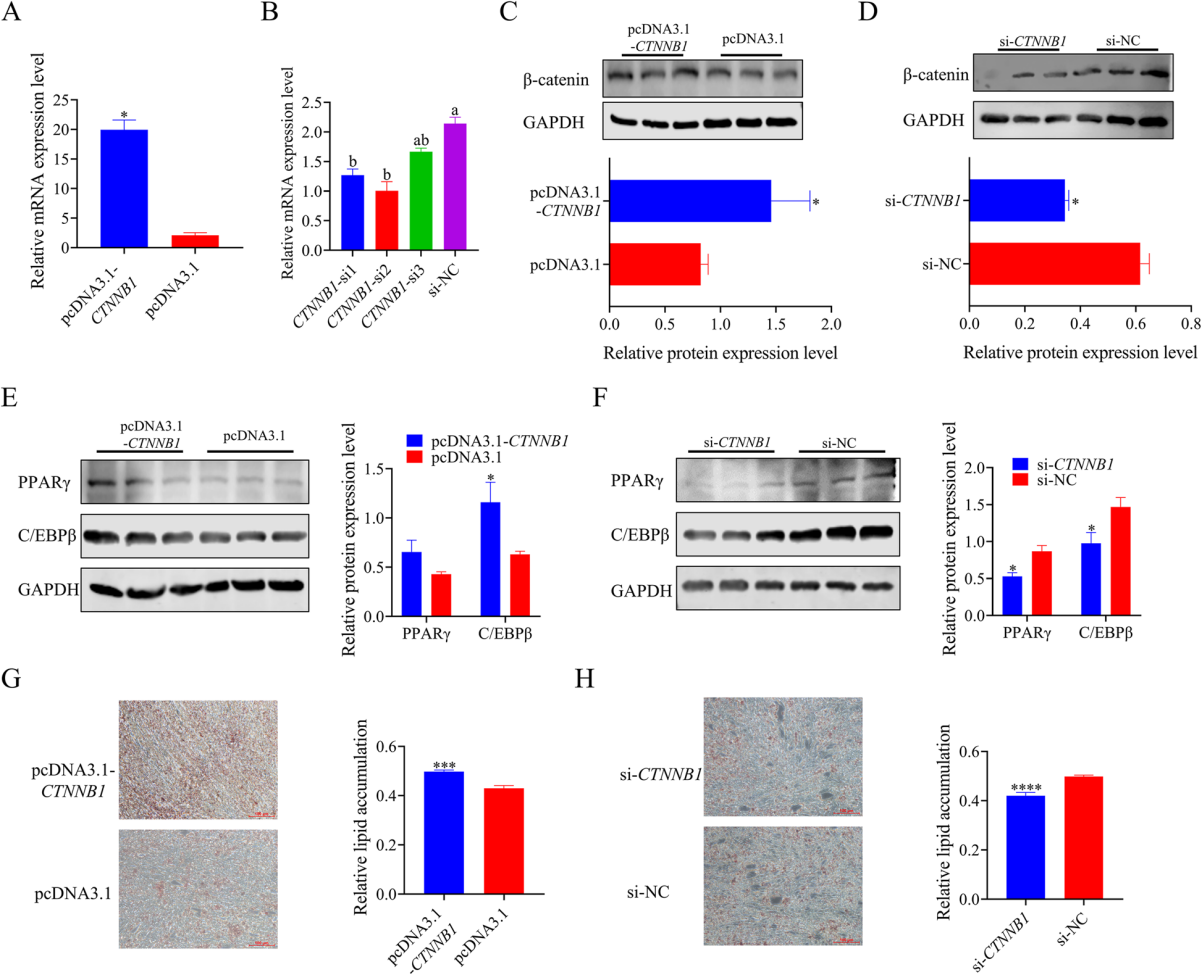
*CTNNB1* promotes lipid accumulation of chicken preadipocytes. **A** and **B** The relative mRNA expression of *CTNNB1* was detected by qPCR with *CTNNB1* overexpressing and knockdown in chicken preadipocytes; **C** and **D** The relative protein expression of β-catenin was detected by western blot; **E** and **F** The relative protein expression of critical differentiation genes was detected with *CTNNB1* overexpression and knockdown in chicken preadipocytes using western blot; **G** and **H** Oil red O staining results and quantification of lipid droplet in chicken preadipocytes after 48 h transfection of *CTNNB1* overexpression plasmid and siRNA. These values are shown as mean ± SEM of at least three biological replicates. The statistical significance of the differences was assessed using the unpaired Student's *t*-test and one-way analysis of variance (ANOVA), followed by Tukey's multiple comparisons test. (*P < 0.05; **P < 0.01; ****P < 0.0001). Groups marked with the same letter were considered to have no significant difference, and those without the same letter were significantly different

To explore the effects of *CTNNB1* on the development of chicken preadipocytes, overexpression plasmid and *CTNNB1* siRNA were constructed and synthesized. RT-PCR assay quantified the relative mRNA expression level of *CTNNB1* after transfection. The results showed that the overexpression and inhibitory effect of *CTNNB1* reached a significant level (Fig. 6A, B). Western blot results also showed that β-catenin protein was significantly promoted and inhibited in chicken preadipocytes (Fig. 6C, D). The protein expression of adipogenic-related genes *C/EBPβ* and *PPARγ* were significantly increased in *CTNNB1*-overexpression cells (Fig. 6E), whereas knockdown of *CTNNB1* resulted in a significant decline (Fig. 6F). Oil red O staining results showed that more lipids were formed when *CTNNB1* overexpression, whereas knockdown of *CTNNB1* dramatically attenuated the lipid accumulation (Fig. 6G, H)**.**

## Discussion

In today's intensive meat production, increased broiler growth rates are often accompanied by increased fat deposition [32]. Excessive fat deposition is energetically wasteful and severely affects poultry carcass quality and feed conversion efficiency [33]. The expansion of adipose tissue was mediated by multiple level regulators, including transcription factors, hormones, dietary factors, and epigenetic modifications [32]. m^6^A is the most common internal modification of eukaryotic RNA and has been reported to play a critical role in the adipogenesis process in pigs and rodents [13, 34]. To examine the relationship between m^6^A methylation levels and lipid deposition in chicken preadipocytes. Our study treated chicken preadipocytes with exogenous methyl donor betaine and methylation inhibitor cycloleucine. Betaine significantly enhanced the global m^6^A level of chicken preadipocytes but at the same time decreased fat deposition, and cycloleucine treatment showed the opposite results, which is in agreement with previous reports. In porcine adipocytes, betaine and cycloleucine modulated m^6^A levels and adipogenesis in a dose-dependent manner [14, 35]. Our results found no difference in cell viability at low concentrations of the betaine treatment group compared with the control group by CCK8 assay; however, flow cytometry EdU assay and RT-PCR showed that betaine appeared to have the same inhibited effect on cell proliferation as cycloleucine. These results seem inconsistent with the changes in m^6^A modification levels they cause. High concentrations of betaine and cycloleucine also showed an inhibitory effect on porcine preadipocytes, which they attributed to the toxicity of the drugs [14, 35]. However, as a natural methyl donor, betaine is involved in multiple epigenetic modifications. Extensive studies have evaluated the effects of betaine on broiler growth and carcass characteristics through the regulation of DNA methylation and histone modifications [36–38]. Therefore, three modifications may be jointly involved in regulating chicken preadipocyte development, resulting in differences in cell viability or gene expression. Interestingly, changes in adipogenesis-related gene expression and lipid accumulation were consistent with changes in m^6^A levels in our study. Therefore betaine and cycloleucine can be used as exogenous compounds to study the regulation of m^6^A level on lipid deposition in poultry. Furthermore, multiple studies have shown that betaine supplementation in the basal diet significantly reduced abdominal adipose deposit and the expression of some lipogenic genes in broilers [39, 40], which is consistent with our results in preadipocytes.

Genome-wide association studies and multiple experimental studies have identified *FTO* as a genetic factor for obesity [15, 16]. Our previous report found that 18 single nucleotide polymorphism sites (SNPs) of the *FTO* gene were significantly associated with chicken body weight and fatness [18]. In addition, since *FTO* was first reported to have demethylase activity efficiently for RNA m^6^A modification in 2011 [22], the demethylation function of *FTO* has been implicated in adipocyte development in multiple mammals. However, its function and mechanism in chicken adipocytes are still unclear. Various *FTO* transcripts were identified in chicken; however, full-length *FTO* was found to be the only transcript in chicken adipose tissue [41]. Our study found that the FTO-NTD domain, which catalyzes demethylase activity, was present in the *FTO* full-length transcript of chicken and other selected species. The full-length transcript of *FTO* significantly decreased the global m^6^A level, and our results also found that *FTO* plays a critical role in adipogenesis by promoting the proliferation rate and lipid accumulation of chicken preadipocytes. Similar results were found in mammals. Knockdown of *FTO* inhibited the proliferation and differentiation of 3T3-L1 preadipocytes [42]. Overexpression of *FTO* in mice could significantly improve body weight and fat mass [43]. Betaine and cycloleucine were found to inhibit and promote *FTO* mRNA expression in 3T3-L1 cells, respectively [44]. The above results indicate that *FTO* is, to a certain extent, a downstream target gene of these two chemicals.

The demethylation activity of FTO on downstream genes has been reported to be associated with adipogenesis. In mouse 3T3-L1 cell line and porcine primary adipocytes, FTO directly mediated autophagy related 5 (*Atg5*) and *Atg7* mRNA expression in an m^6^A dependent manner, thereby adipogenesis and autophagy [13]. Loss of *FTO* significantly decreased the expression of cyclin A2 (*CCNA2*) and cyclin-dependent kinase 2 (*CDK2*) mRNA by increasing their m^6^A levels, thereby delaying cell cycle progression and inhibiting adipogenesis in 3T3-L1 cells [24]. Metformin increases m^6^A methylation levels of *CCND1* and *CDK2* by inhibiting FTO expression, thereby inhibiting adipogenesis and combating obesity [25]. In our study, overexpression and knockdown of *FTO* markedly altered the mRNA and protein expression levels of *CTNNB1* in an m^6^A modification manner. The most commonly implicated Wnt pathway is through β-catenin, a classical and critical core factor of Wnt signaling [45]. It is well known that the Wnt/β-catenin signaling pathway usually maintains cell stemness and determines cell fate [46]. In adipose tissue, Wnt/β-catenin signaling mainly maintains the stemness of preadipocytes and plays an essential role in the directed differentiation of mesenchymal stem cells (MCE) into adipocytes [47]. Interestingly, most current related studies were focused on the early stage of adipogenesis, consistently with our findings. The knockdown of *FTO* did not affect adipogenic gene expression after adipogenic induction [43].

Multiple previous studies identified Wnt/β-catenin signaling as a critical inhibitor of adipocyte differentiation [48, 49]. Interestingly, a recent report found that *CTNNB1* expression was significantly higher in obese mice than in normal-weight mice; and ablation of *CTNNB1* in mice mature adipocytes reduced macrophage expansion, thereby decreasing inguinal white adipose tissue expansion and obesity caused by overnutrition [50]. ICP cells used in our study are stromal vascular fraction (SVF) cells isolated from chicken adipose tissue by immortalization [51]; cell types are similar to the mouse mature adipocyte types reported above. Moreover, the addition of oleic acid, an essential exogenous fatty acid for chicken adipocyte differentiation, appears to mimic the overnutrition conditions in mice. In the current study, our results also found that *CTNNB1* knockdown attenuated lipid accumulation of chicken preadipocytes compared to the control group, and overexpression of *CTNNB1* showed the opposite results. In vivo and in vitro experiments in mice showed that deletion of *CTNNB1* resulted in reduced expression of critical de novo lipogenesis genes, including *SREBF1*, ATP citrate lyase, acetyl-CoA carboxylase ACC1, fatty acid synthase, and stearoyl-Coenzyme A desaturase 1 [47]. The synthesis of fatty acids in poultry is mainly in the liver, accounting for about 90%, but the adipogenesis ability of adipocytes is weak [4]. Perhaps the attenuated expression of lipogenic genes caused by *CTNNB1* ablation further reduces lipid accumulation in chicken preadipocytes. Furthermore, a small-molecule inhibitor, iCRT14, could dose-dependently inhibit adipogenesis of human stromovascular cells by selectively attenuating β-catenin transcription activity [52]. These may explain that chicken *CTNNB1* knockdown led to less lipogenesis.

## Conclusions

Our findings unraveled that the RNA m^6^A methylation levels were negatively correlated with adipogenesis in chicken preadipocytes, and FTO could promote the proliferation and differentiation capacity of chicken preadipocytes in vitro. Mechanistically, FTO directly targeted *CTNNB1* transcripts in an m^6^A-demethylation manner to regulate its mRNA expression, thereby affecting adipogenesis. Our work provides new insights into the critical roles of m^6^A methylation in chicken adipogenesis and will facilitate the development of further research.

## Supplementary Information

**Additional file 1: Table S1.** Information of primers. **Table S2.** Oligonucleotides. **Table S3.** The SELECT primer. **Table S4.** SRAMP prediction results of *CTNNB1* m^6^A sites. **Fig. S1.** The cell proliferation analysis and Oil red O staining were treated with betaine and cycloleucine. **Fig. S2.** The Nile red staining results were treated with betaine and cycloleucine. **Fig. S3.** The prediction results of PRIdictor. **Fig. S4.** The SELECT results of sites 420 and 2816 site with *FTO* knockdown preadipocytes.

## Data Availability

The data were exhibited in the main manuscript and supplemental materials.

## Abbreviations

m^6^A: N6-methyladenosine
FTO: The fat mass and obesity-associated
ICP: Immortalized chicken preadipocytes
GWAS: Genome-wide associated studies
SMART: Simple modular architecture research tool
RIPA: Radio immunoprecipitation assay
PMSF: Phenyl methyl sulfonyl fluoride
SDS-PAGE: Polyacrylamide gel electrophoresis
PVDF: Polyvinylidene difluoride
TBST: Pris buffered saline with Tween
CCK-8: Cell counting Kit 8
EdU: 5-Ethynyl-2’-Deoxyuridine assay
SELECT: Single-base elongation and ligation-based PCR amplification method
MeRIP: Methylated RNA immunoprecipitation assay
RIP: RNA-binding protein immunoprecipitation assay
SAM: S-adenosylmethionine

## References

[1] Kopelman PG (2000). Obesity as a medical problem. Nature.

[2] Kwok S, Adam S, Ho JH, Iqbal Z, Turkington P, Razvi S (2020). Obesity: a critical risk factor in the COVID-19 pandemic. Clin Obes.

[3] Livingstone M (2001). Childhood obesity in Europe: a growing concern. Public Health Nutr.

[4] Fouad A, El-Senousey H (2014). Nutritional factors affecting abdominal fat deposition in poultry: a review. Asian-Australa J Anim Sci.

[5] Claire D’Andre H, Paul W, Shen X, Jia X, Zhang R, Sun L, et al. Identification and characterization of genes that control fat deposition in chickens. J Animal Sci Biotechnol. 2013;4:43. 10.1186/2049-1891-4-43. 10.1186/2049-1891-4-43 PMC3874612 24206759

[6] Reaven GM (1999). Insulin resistance: a chicken that has come to roost. Ann N Y Acad Sci.

[7] Rosen ED, Spiegelman BM (2000). Molecular regulation of adipogenesis. Annu Rev of cell Dev Biol.

[8] Nematbakhsh S, Pei Pei C, Selamat J, Nordin N, Idris LH, Abdull Razis AF (2021). Molecular regulation of lipogenesis, adipogenesis and fat deposition in chicken. Genes.

[9] Pant R, Firmal P, Shah VK, Alam A, Chattopadhyay S. Epigenetic regulation of adipogenesis in development of metabolic syndrome. Front Cell Dev Biol. 2021;8:619888. 10.3389/fcell.2020.619888. 10.3389/fcell.2020.619888 PMC7835429 33511131

[10] Wu J, Frazier K, Zhang J, Gan Z, Wang T, Zhong X. Emerging role of m^6^A RNA methylation in nutritional physiology and metabolism. Obes Rev. 2020;21(1):e12942. 10.1111/obr.12942. 10.1111/obr.12942 PMC7427634 31475777

[11] Yang C, Hu Y, Zhou B, Bao Y, Li Z, Gong C (2020). The role of m6A modification in physiology and disease. Cell Death Dis.

[12] Meyer KD, Jaffrey SR (2017). Rethinking m6A readers, writers, and erasers. Ann Review Cell Dev Biol.

[13] Wang X, Wu R, Liu Y, Zhao Y, Bi Z, Yao Y (2020). m6A mRNA methylation controls autophagy and adipogenesis by targeting Atg5 and Atg7. Autophagy.

[14] Wang X, Zhu L, Chen J, Wang Y (2015). mRNA m6A methylation downregulates adipogenesis in porcine adipocytes. Biochem Biophys Res Commun.

[15] Dina C, Meyre D, Gallina S, Durand E, Körner A, Jacobson P (2007). Variation in FTO contributes to childhood obesity and severe adult obesity. Nat Genet.

[16] Frayling TM, Timpson NJ, Weedon MN, Zeggini E, Freathy RM, Lindgren CM (2007). A common variant in the FTO gene is associated with body mass index and predisposes to childhood and adult obesity. Science.

[17] Scuteri A, Sanna S, Chen W-M, Uda M, Albai G, Strait J (2007). Genome-wide association scan shows genetic variants in the FTO gene are associated with obesity-related traits. PLoS Genet.

[18] Jia X, Nie Q, Lamont S, Zhang X (2012). Variation in sequence and expression of the avian FTO, and association with glucose metabolism, body weight, fatness and body composition in chickens. Int J Obes.

[19] McMurray F, Church CD, Larder R, Nicholson G, Wells S, Teboul L (2013). Adult onset global loss of the fto gene alters body composition and metabolism in the mouse. PLoS Genet.

[20] Fischer J, Koch L, Emmerling C, Vierkotten J, Peters T, Brüning JC (2009). Inactivation of the Fto gene protects from obesity. Nature.

[21] Church C, Moir L, McMurray F, Girard C, Banks GT, Teboul L (2010). Overexpression of Fto leads to increased food intake and results in obesity. Nat Genet.

[22] Jia G, Fu Y, Zhao X, Dai Q, Zheng G, Yang Y (2011). N 6-methyladenosine in nuclear RNA is a major substrate of the obesity-associated FTO. Nat Chem Biol.

[23] Zhao X, Yang Y, Sun B-F, Shi Y, Yang X, Xiao W (2014). FTO-dependent demethylation of N6-methyladenosine regulates mRNA splicing and is required for adipogenesis. Cell Res.

[24] Wu R, Liu Y, Yao Y, Zhao Y, Bi Z, Jiang Q (2018). FTO regulates adipogenesis by controlling cell cycle progression via m6A-YTHDF2 dependent mechanism. Biochim Biophys Acta Mol Cell Bio Lipids.

[25] Liao X, Liu J, Chen Y, Liu Y, Chen W, Zeng B, et al. Metformin combats obesity by targeting FTO in an m6A-YTHDF2-dependent manner. J Drug Target. 2022;30(2):219–31. 10.1080/1061186X.2021.1961790. 10.1080/1061186X.2022.2071906 35481401

[26] Xiao Y, Wang Y, Tang Q, Wei L, Zhang X, Jia G (2018). An elongation-and ligation-based qPCR amplification method for the radiolabeling-free detection of locus-specific N6-methyladenosine modification. Angew Chem Int Ed Engl.

[27] Purohit V, Abdelmalek MF, Barve S, Benevenga NJ, Halsted CH, Kaplowitz N (2007). Role of S-adenosylmethionine, folate, and betaine in the treatment of alcoholic liver disease: summary of a symposium. Am J Clin Nutr.

[28] Zhou Y, Zeng P, Li Y-H, Zhang Z, Cui Q (2016). SRAMP: prediction of mammalian N6-methyladenosine (m6A) sites based on sequence-derived features. Nucleic Acids Res..

[29] Cheng B, Leng L, Li Z, Wang W, Jing Y, Li Y (2021). Profiling of RNA N6-Methyladenosine methylation reveals the critical role of m6A in chicken adipose deposition. Front Cell Dev Biol.

[30] Muppirala UK, Honavar VG, Dobbs D (2011). Predicting RNA-protein interactions using only sequence information. BMC Bioinformatics.

[31] Tuvshinjargal N, Lee W, Park B, Han K (2016). PRIdictor: protein–RNA interaction predictor. Biosystems.

[32] Wang G, Kim WK, Cline MA, Gilbert ER (2017). Factors affecting adipose tissue development in chickens: A review. Poult Sci.

[33] Hausman GJ, Bergen WG, Etherton TD, Smith SB (2018). The history of adipocyte and adipose tissue research in meat animals. J Anim Sci.

[34] Wu R, Guo G, Bi Z, Liu Y, Zhao Y, Chen N (2019). m6A methylation modulates adipogenesis through JAK2-STAT3-C/EBPβ signaling. Biochim Biophys Acta Gene Regul Mech.

[35] Kang H, Zhang Z, Yu L, Li Y, Liang M, Zhou L (2018). FTO reduces mitochondria and promotes hepatic fat accumulation through RNA demethylation. J Cell Biochem.

[36] Idriss AA, Hu Y, Sun Q, Jia L, Jia Y, Omer NA (2017). Prenatal betaine exposure modulates hypothalamic expression of cholesterol metabolic genes in cockerels through modifications of DNA methylation. Poult Sci.

[37] Yang S, Zhao N, Sun B, Yang Y, Hu Y, Zhao R (2020). Grandmaternal betaine supplementation enhances hepatic IGF2 expression in F2 rat offspring through modification of promoter DNA methylation. J Sci Food Agric.

[38] Sternbach S, West N, Singhal NK, Clements R, Basu S, Tripathi A (2021). The BHMT-betaine methylation pathway epigenetically modulates oligodendrocyte maturation. PLoS ONE.

[39] Esteve-Garcia E, Mack S (2000). The effect of DL-methionine and betaine on growth performance and carcass characteristics in broilers. Anim Feed Sci Tech.

[40] McDevitt R, Mack S, Wallis I (2000). Can betaine partially replace or enhance the effect of methionine by improving broiler growth and carcase characteristics?. Br Poult Sci.

[41] Tiwari A, Krzysik-Walker S, Ramachandran R (2012). Cloning and characterization of chicken fat mass and obesity associated (Fto) gene: fasting affects Fto expression. Domest Anim Endocrinol.

[42] Jiao Y, Zhang J, Lu L, Xu J, Qin L (2016). The Fto gene regulates the proliferation and differentiation of pre-adipocytes in vitro. Nutrients.

[43] Merkestein M, Laber S, McMurray F, Andrew D, Sachse G, Sanderson J (2015). FTO influences adipogenesis by regulating mitotic clonal expansion. Nat Commun.

[44] Shen Z, Liu P, Sun Q, Li Y, Acharya R, Li X (2021). FTO inhibits UPRmt-induced apoptosis by activating JAK2/STAT3 pathway and reducing m6A level in adipocytes. Apoptosis.

[45] Clevers H, Nusse R (2012). Wnt/β-catenin signaling and disease. Cell.

[46] De Winter TJ, Nusse R (2021). Running against the Wnt: How Wnt/β-catenin suppresses adipogenesis. Front Cell Dev Bio.

[47] Bagchi DP, Nishii A, Li Z, DelProposto JB, Corsa CA, Mori H (2020). Wnt/β-catenin signaling regulates adipose tissue lipogenesis and adipocyte-specific loss is rigorously defended by neighboring stromal-vascular cells. Mol Metab.

[48] Christodoulides C, Lagathu C, Sethi JK, Vidal-Puig A (2009). Adipogenesis and WNT signalling. Trends Endocrinol Metab.

[49] Ross SE, Hemati N, Longo KA, Bennett CN, Lucas PC, Erickson RL (2000). Inhibition of adipogenesis by Wnt signaling. Science.

[50] Chen M, Lu P, Ma Q, Cao Y, Chen N, Li W (2020). CTNNB1/β-catenin dysfunction contributes to adiposity by regulating the cross-talk of mature adipocytes and preadipocytes. Sci Adv..

[51] Wang W, Zhang T, Wu C, Wang S, Wang Y, Li H (2017). Immortalization of chicken preadipocytes by retroviral transduction of chicken TERT and TR. PLoS ONE.

[52] Loh NY, Neville MJ, Marinou K, Hardcastle SA, Fielding BA, Duncan EL (2015). LRP5 regulates human body fat distribution by modulating adipose progenitor biology in a dose-and depot-specific fashion. Cell Metab.

