# Supplementary material for: m6A demethylase FTO regulate CTNNB1 to promote adipogenesis of chicken preadipocyte
Source: J Anim Sci Biotechnol. 2022 Dec 2;13:147. doi: 10.1186/s40104-022-00795-z (PMC9716549; doi:10.1186/s40104-022-00795-z)
Supplement: Supplementary file 1 — Additional file 1: Table S1. Information of primers. Table S2. Oligonucleotides. Table S3. The SELECT primer. Table S4. SRAMP prediction results of CTNNB1 m6A sites. Fig. S1. The cell proliferation analysis and Oil red O staining were treated with betaine and cycloleucine. Fig. S2. The Nile red staining results were treated with betaine and cycloleucine. Fig. S3. The prediction results of PRIdictor. Fig. S4. The SELECT results of sites 420 and 2816 site with FTO knockdown preadipocytes. [file 40104_2022_795_MOESM1_ESM.docx]

| Primer | Primer sequence | Annealing temperature, | Product size |
| --- | --- | --- | --- |
| *PCNA* | F: GTGCTGGGACCTGGGTT  R: CGTATCCGCATTGTCTTCT | 58 | 217 |
| *CCNB2* | F: CAGTAAAGGCTACGAAAG  R: ACATCCATAGGGACAGG | 58 | 133 |
| *CCND1* | F: CAGAAGTGCGAAGAGGAAGT  R: CTGATGGAGTTGTCGGTGTA | 58 | 188 |
| *CCND2* | F: AACTTGCTCTACGACGACC  R: TTCACAGACCTCCAACATC | 58 | 150 |
| *C/EBPα* | F: CTCCTCACGCTTTGGTAA  R: TGATAGTCGTATGGGTTGGT | 58 | 213 |
| *C/EBPβ* | F: CGGAGGCTGAAGAAGGTGAA  R: CGGTCCTCTGCCTGGTCAT | 58 | 320 |
| *PPARγ* | F: GCTACTACACGGAATCACCAAAT  R: CTGGGCTCCACTGTCACTCA | 58 | 200 |
| *GAPDH* | F: TCCTCCACCTTTGATGCG  R: GTGCCTGGCTCACTCCTT | 58 | 146 |
| pcDNA3.1-*FTO* | F: **GCTAGC**ATGAAGAGGAGAGCAGGGGA | Vector construction | |
|  | R: **GATATC**CTACTGAACAAGATTTTGTA |  |  |
| pcDNA3.1-*CTNNB1* | F: **GGATCC**ATGGCAACCCAAGCTGACTT | Vector construction | |
|  | R: **CTCGAG**CAGGTCAGTATCGAACCAGG |  |  |
| pcDNA3.1-*FTO*-3×Flag | F: **GGATCC**ATGAAGAGGAGAGCAGGGGAGC | Vector construction | |
|  | R: **GAATTC**CTGAACAAGATTTTGTAGATGAA |  |  |
| MeRIP-qPCR primers | |  |  |
| 420 site | F: ATGGCAACCCAAGCTGACTT | 58 | 164 |
|  | R: CTTGCGTTGTGTCCACATC |  |  |
| 2816 site | F: CTCGAGGGGTGGGCTAGTAT | 58 | 154 |
|  | R: TCCTCGTTTCTTGAATCACT |  |  |

**Table S1** Information of primers

Sequences in bold represent the enzyme cutting sites

**Table S2** Oligonucleotides

| Fragment name | Sequences (5’ to 3’) |
| --- | --- |
| si-*FTO* | CCAGAUAUUCCAAGCUAAUTT |
|  | AUUAGCUUGGAAUAUCUGGTT |
| *CTNNB1*- si1 | GCUGAUAUUGAUGGUCAAUTT |
|  | AUUGACCAUCAAUAUCAGCTT |
| *CTNNB1*- si2 | GCUUUAGGACUCCACCUUATT |
|  | UAAGGUGGAGUCCUAAAGCTT |
| *CTNNB1*- si3 | CCCUAUGAUGGAACAUGAATT |
|  | UUCAUGUUCCAUCAUAGGGTT |
| si-NC | UUCUCCGAACGUGUCACGUTT |
|  | ACGUGACACGUUCGGAGAATT |

**Table S3** The SELECT primer

| Primer | Sequences (5’ to 3’) |
| --- | --- |
| *CTNNB1* 420 X-site UP probe | tagccagtaccgtagtgcgtgCACCGGAATGGATACCAGAG |
| *CTNNB1* 420 site down probe | CCAGATATGACTGCTGCTGCcagaggctgagtcgctgcat |
| *CTNNB1* 420 N-site UP probe | tagccagtaccgtagtgcgtgAATGGATACCAGAGTCCAGA |
| *CTNNB1* 420 N-site down probe | ATGACTGCTGCTGCCAATGAcagaggctgagtcgctgcat |
| *CTNNB1* 2816 X-site UP probe | tagccagtaccgtagtgcgtgAAAAGGACCAGAACAAAAAG |
| *CTNNB1* 2816 site down probe | TTACTTCAATTGTTCCCATAcagaggctgagtcgctgcat |
| *CTNNB1* 2816 N-site UP probe | tagccagtaccgtagtgcgtgACCAGAACAAAAAGTTTACT |
| *CTNNB1* 2816 N-site down probe | CAATTGTTCCCATAGGAAACcagaggctgagtcgctgcat |
| Select qPCR forward primer | ATGCAGCGACTCAGCCTCTG |
| Select qPCR reverse primer | TAGCCAGTACCGTAGTGCGTG |

**Table S4** SRAMP prediction results of *CTNNB1* m^6^A sites

| Position | Sequence context | Score (binary) | Score (knn) | Score (spectrum) | Score (combined) | Decision |
| --- | --- | --- | --- | --- | --- | --- |
| 420 | GGACU | 0.713 | 0.719 | 0.510 | 0.632 | High confidence |
| 498 | GGACA | 0.588 | 0.501 | 0.546 | 0.567 | Low confidence |
| 790 | GGACU | 0.674 | 0.592 | 0.429 | 0.572 | Low confidence |
| 1221 | AGACU | 0.720 | 0.679 | 0.538 | 0.645 | High confidence |
| 1464 | GAACU | 0.695 | 0.728 | 0.494 | 0.616 | Moderate confidence |
| 1475 | GGACC | 0.552 | 0.498 | 0.572 | 0.557 | Low confidence |
| 1795 | GGACU | 0.643 | 0.641 | 0.566 | 0.612 | Moderate confidence |
| 2185 | GAACU | 0.617 | 0.638 | 0.487 | 0.566 | Low confidence |
| 2356 | GAACU | 0.653 | 0.686 | 0.594 | 0.631 | High confidence |
| 2378 | GGACU | 0.710 | 0.458 | 0.653 | 0.674 | High confidence |
| 2416 | GGACU | 0.715 | 0.594 | 0.572 | 0.651 | High confidence |
| 2520 | GGACC | 0.699 | 0.663 | 0.506 | 0.620 | High confidence |
| 2533 | GAACA | 0.671 | 0.293 | 0.583 | 0.617 | Moderate confidence |
| 2701 | GAACU | 0.616 | 0.49 | 0.561 | 0.587 | High confidence |
| 2816 | AAACU | 0.801 | 0.333 | 0.710 | 0.641 | Very high confidence |


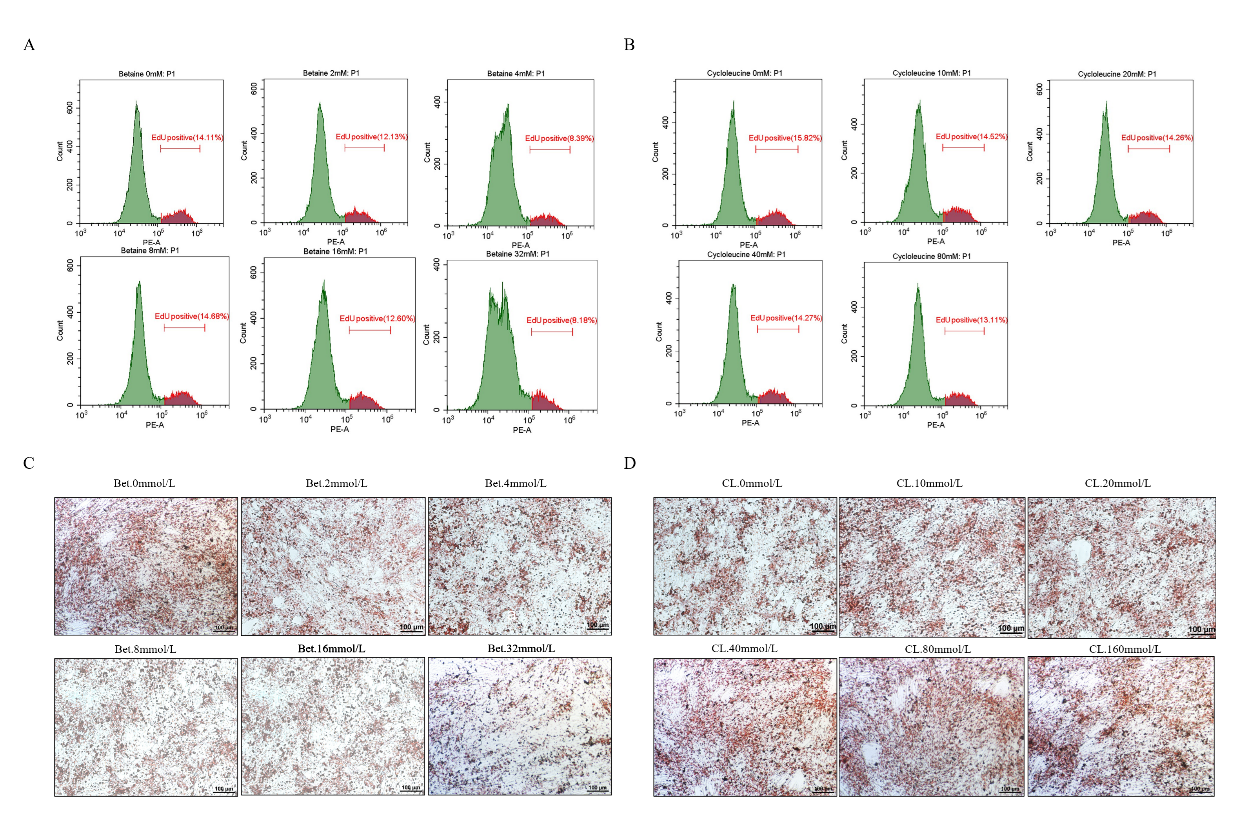


**Fig. S1** The cell proliferation analysis and Oil red O staining were treated with betaine and cycloleucine. **(A and B)** The percent of EdU positive cells analysis by flow cytometry EdU assay in chicken treated with different doses of betaine and cycloleucine. **(C and D)** The images of Oil red O staining of betaine and cycloleucine treatment in chicken preadipocyte


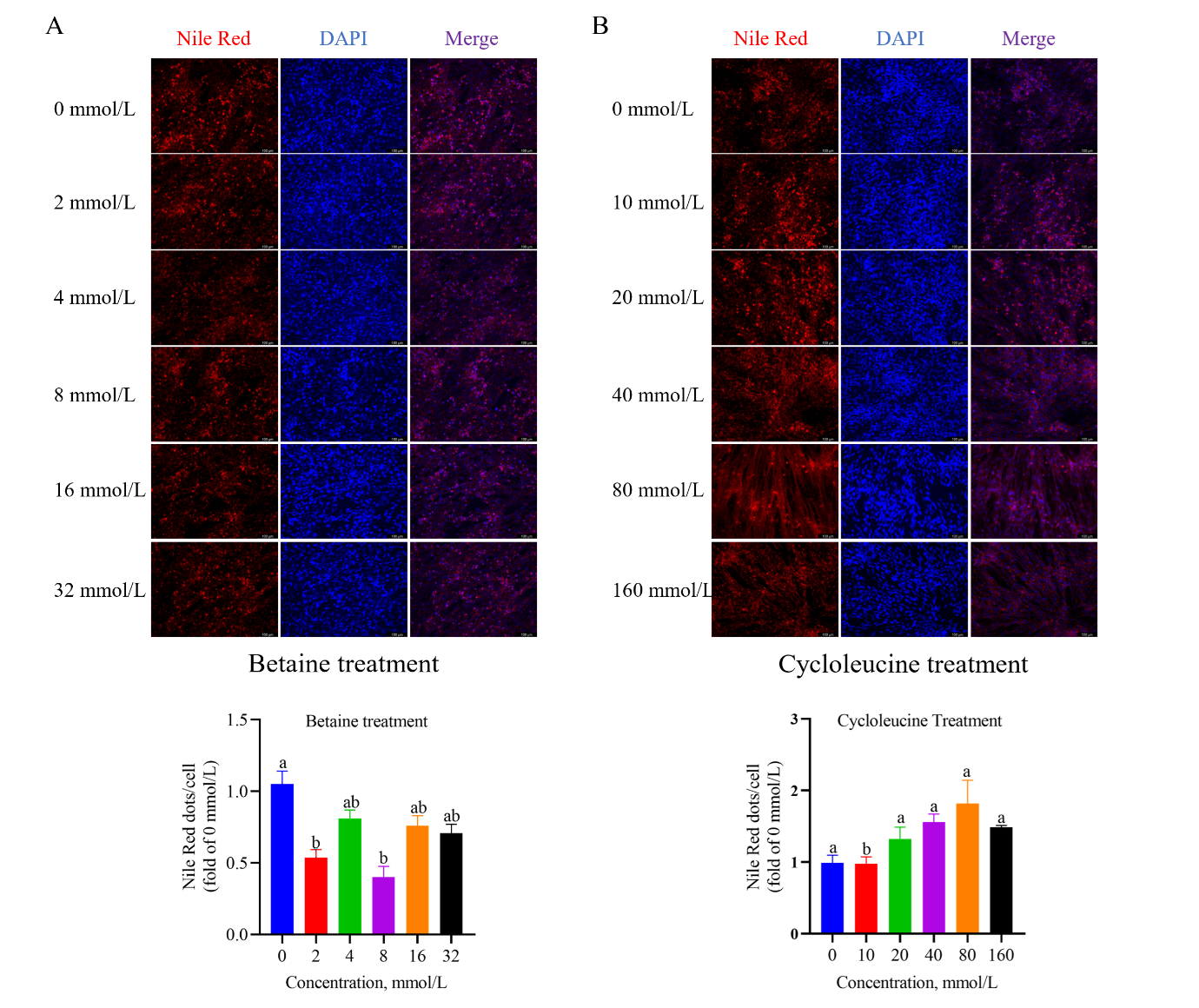


**Fig. S2** The Nile red staining results were treated with betaine and cycloleucine. **(A)** The images of Nile red (lipid) and DAPI (nuclei) staining and the normalized results of adipocytes treated with different doses of betaine. **(B)** The images of Nile red (lipid) and DAPI (nuclei) staining and the normalized results of adipocytes treated with different doses of cycloleucine


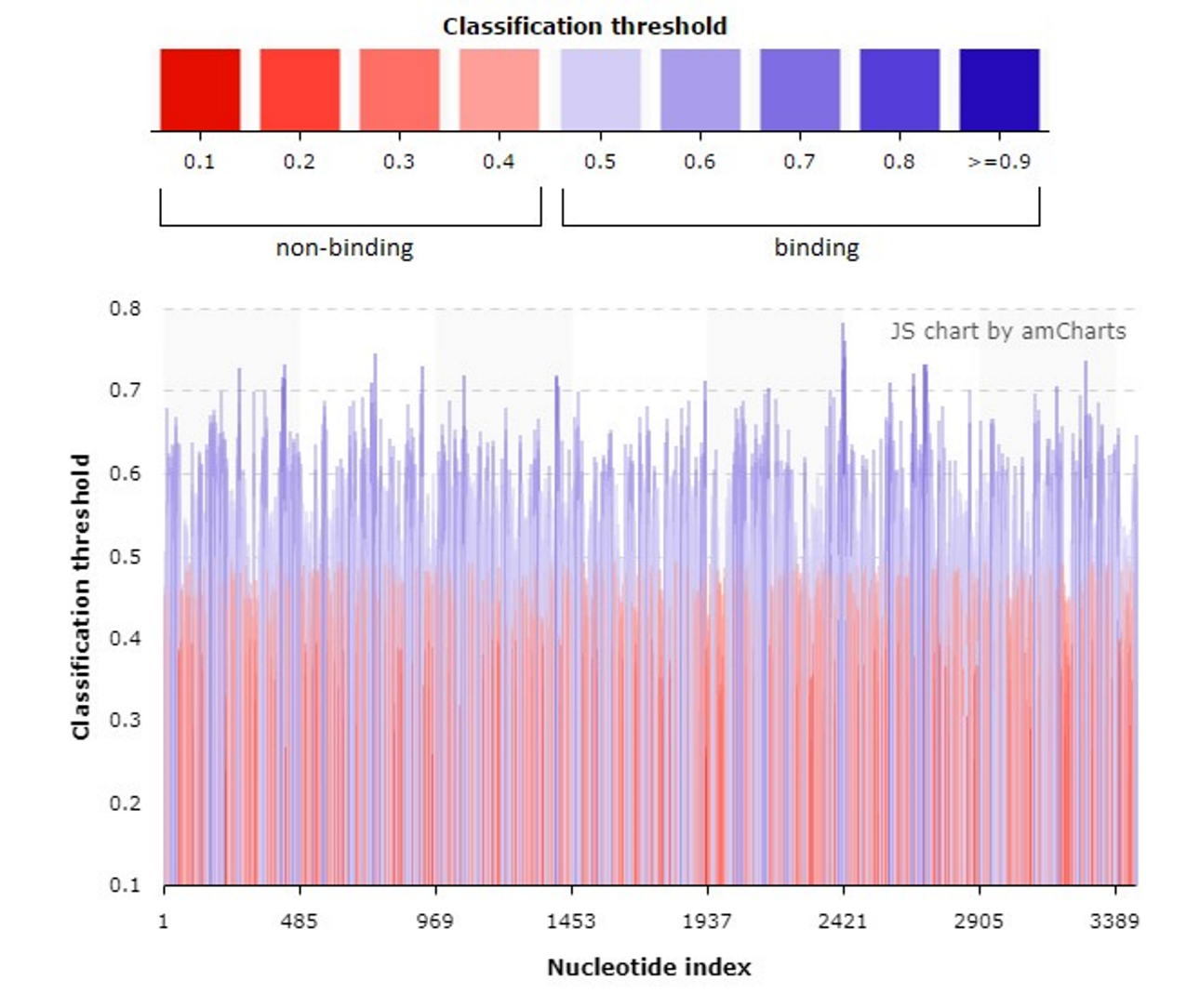


**Fig. S3** The prediction results of PRIdictor. Predicted binging sites with the classification threshold value of 0.5


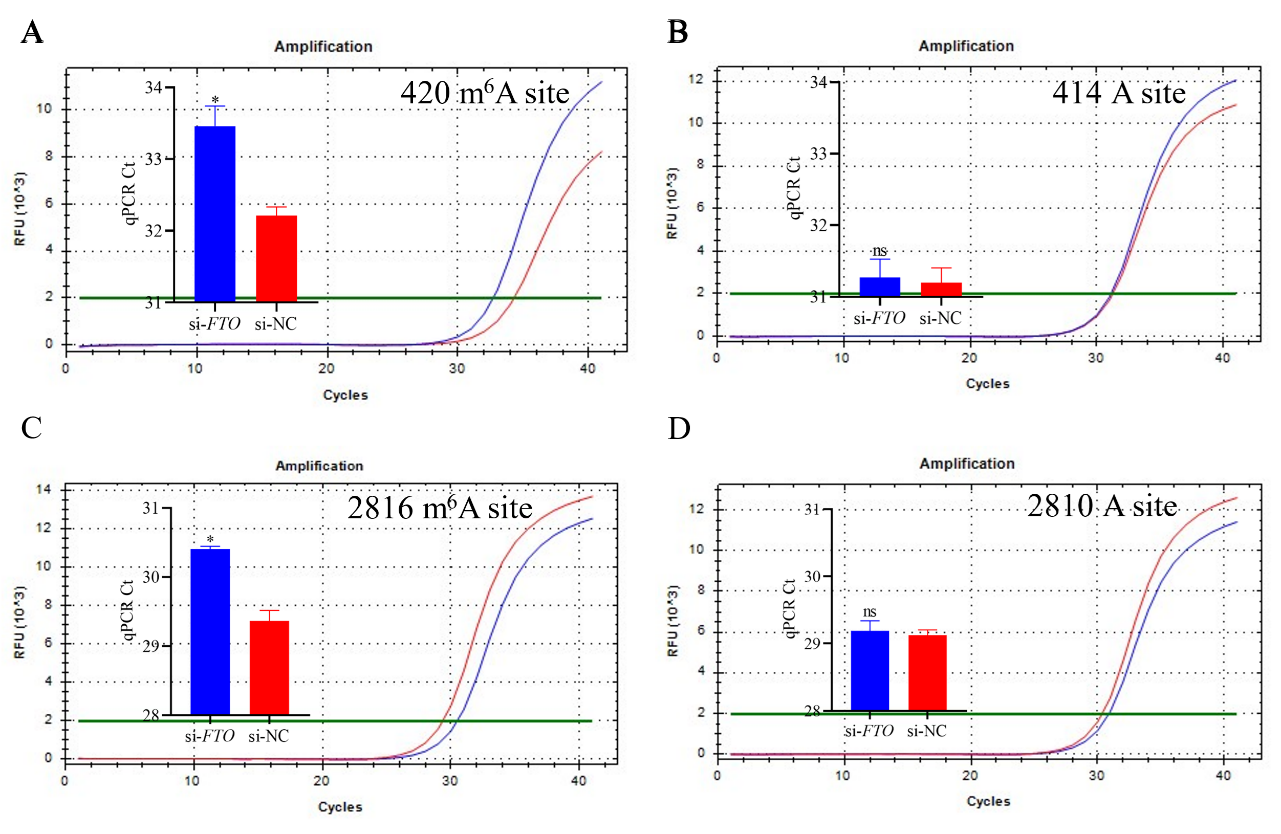


**Fig. S4** The SELECT results of sites 420 and 2816 site with *FTO* knockdown preadipocytes. **(A and B)** Amplification curve and qPCR CT value in *CTNNB1* 420 m^6^A site and 414 A site after *FTO* knockdown; **(C and D)** Amplification curve and qPCR CT value in *CTNNB1* 2816 m^6^A site and 2810 A site after *FTO* knockdown. These values are shown as mean ± (SEM) of at least three biological replicates. The statistical significance of the differences was assessed using the unpaired Student's *t*-test (**P*< 0.05)
